# Using convolutional neural networks to count parrot nest‐entrances on photographs from the largest known colony of Psittaciformes

**DOI:** 10.1002/ece3.70172

**Published:** 2024-08-13

**Authors:** Gabriel L. Zanellato, Gabriel A. Pagnossin, Mauricio Failla, Juan F. Masello

**Affiliations:** ^1^ Fundación Soberanía Cinco Saltos Río Negro Argentina; ^2^ Universidad Nacional de Río Negro General Roca Río Negro Argentina; ^3^ Proyecto Patagonia Noreste Balneario El Cóndor Río Negro Argentina; ^4^ Department of Evolutionary Population Genetics Bielefeld University Bielefeld Germany; ^5^ Department of Biological Sciences University of Venda Thohoyandou South Africa

**Keywords:** artificial intelligence, burrow nesting, colony, computer vision, convolutional neural networks, machine learning, object counting

## Abstract

Counting animal populations is fundamental to understand ecological processes. Counts make it possible to estimate the size of an animal population at specific points in time, which is essential information for understanding demographic change. However, in the case of large populations, counts are time‐consuming, particularly if carried out manually. Here, we took advantage of convolutional neural networks (CNN) to count the total number of nest‐entrances in 222 photographs covering the largest known Psittaciformes (Aves) colony in the world. We conducted our study at the largest Burrowing Parrot *Cyanoliseus patagonus* colony, located on a cliff facing the Atlantic Ocean in the vicinity of El Cóndor village, in north‐eastern Patagonia, Argentina. We also aimed to investigate the distribution of nest‐entrances along the cliff with the colony. For this, we used three CNN architectures, U‐Net, ResUnet, and DeepLabv3. The U‐Net architecture showed the best performance, counting a mean of 59,842 Burrowing Parrot nest‐entrances across the colony, with a mean absolute error of 2.7 nest‐entrances over the testing patches, measured as the difference between actual and predicted counts per patch. Compared to a previous study conducted at El Cóndor colony more than 20 years ago, the CNN architectures also detected noteworthy differences in the distribution of the nest‐entrances along the cliff. We show that the strong changes observed in the distribution of nest‐entrances are a measurable effect of a long record of human‐induced disturbance to the Burrowing Parrot colony at El Cóndor. Given the paramount importance of the Burrowing Parrot colony at El Cóndor, which concentrates 71% of the world's population of this species, we advocate that it is imperative to reduce such a degree of disturbance before the parrots reach the limit of their capacity of adaptation.

## Graphical abstract

We used a convolutional neural network computer vision technique to count the number of nest‐entrances at the El Cóndor Burrowing Parrot colony. El Cóndor colony is of utmost importance for the conservation of the species: 71% of the global population of burrowing parrots breeds there. Any threats affecting the El Cóndor colony could utterly impact the whole of the species. This colony is also deemed the largest known Psittaciformes colony in the world. In our study, we detected noteworthy differences in the distribution of the nest‐entrances, and showed that this is a measurable effect of human‐induced disturbance.

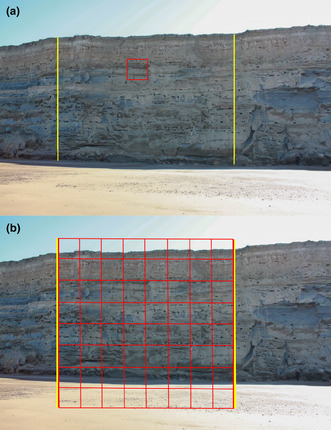

## 1 INTRODUCTION

Quantifying animal populations provides information that is fundamental to understand ecological and evolutionary processes and mechanisms (Liu et al., 2020; Robinson et al., 2022; Volkov et al., 2003). It is also a basic requirement for ascertaining the relative frequency of a species within a community, its population trend, and conservation status (Eikelboom et al., 2019; Keeping & Pelletier, 2014; Volkov et al., 2003). Knowing the abundance of a species over time will help to understand changes in natural mortality, the effects of environmental change on survival, the impact of the current degree of anthropogenic environmental change, identify threats, and facilitate effective conservation measures (Brlík et al., 2021; Dénes et al., 2015; Kellenberger et al., 2021; Ripple et al., 2022).

Numerous animal species, including many birds, breed in large colonies (Ainley et al., 2003; Liu et al., 2020; Masello et al., 2006). A number of techniques have been used to estimate their population sizes, including ground‐based surveys, counts from photographs and satellite images, and the use of camera traps and unoccupied aerial vehicles (Bibby et al., 1993; Boersma, 1998; Dickens et al., 2021; Fretwell & Trathan, 2021; Masello et al., 2006; Santangeli et al., 2022; Suwanrat et al., 2015). All those methods have advantages and limitations with respect to practicability, costs, or disturbance to wildlife, but a common feature is that the manual counts involved are typically time‐consuming (Bowler et al., 2020; Kellenberger et al., 2021; Wang et al., 2019). Nowadays, the increasing use of image analysis techniques is facilitating more efficient wildlife counts (Acevedo & Villanueva‐Rivera, 2006; Grenzdörffer, 2013; Hollings et al., 2019; Youngflesh et al., 2021). Some supervised machine learning algorithms, particularly those capable of regressing a density map, enable the detection of a large number of individuals from images with reduced manual effort and time investment (Fiaschi et al., 2012; Lempitsky & Zisserman, 2010; Xie et al., 2018). Such machine‐learning algorithms are trained by providing them with samples of desired input‐output pairs, rather than the more complex task of programming rules to anticipate responses for every possible input. These inputs are patches of the original images in which we need to count objects, and the outputs are density maps corresponding to the input patches (Lempitsky & Zisserman, 2010). Convolutional neural networks (CNN) are designed to process pixel data and thus, can process data organized in multiple arrays like colour photographs, which contain pixel intensities in the three colour channels (reviewed in LeCun et al., 2015; Dujon & Schofield, 2019). CNN have been successfully used to count birds from images in recent years (Akçay et al., 2020; Borowicz et al., 2018; Bowler et al., 2020; Hong et al., 2019; Kellenberger et al., 2021; Liu et al., 2020; Wang et al., 2023).

**FIGURE 1:**
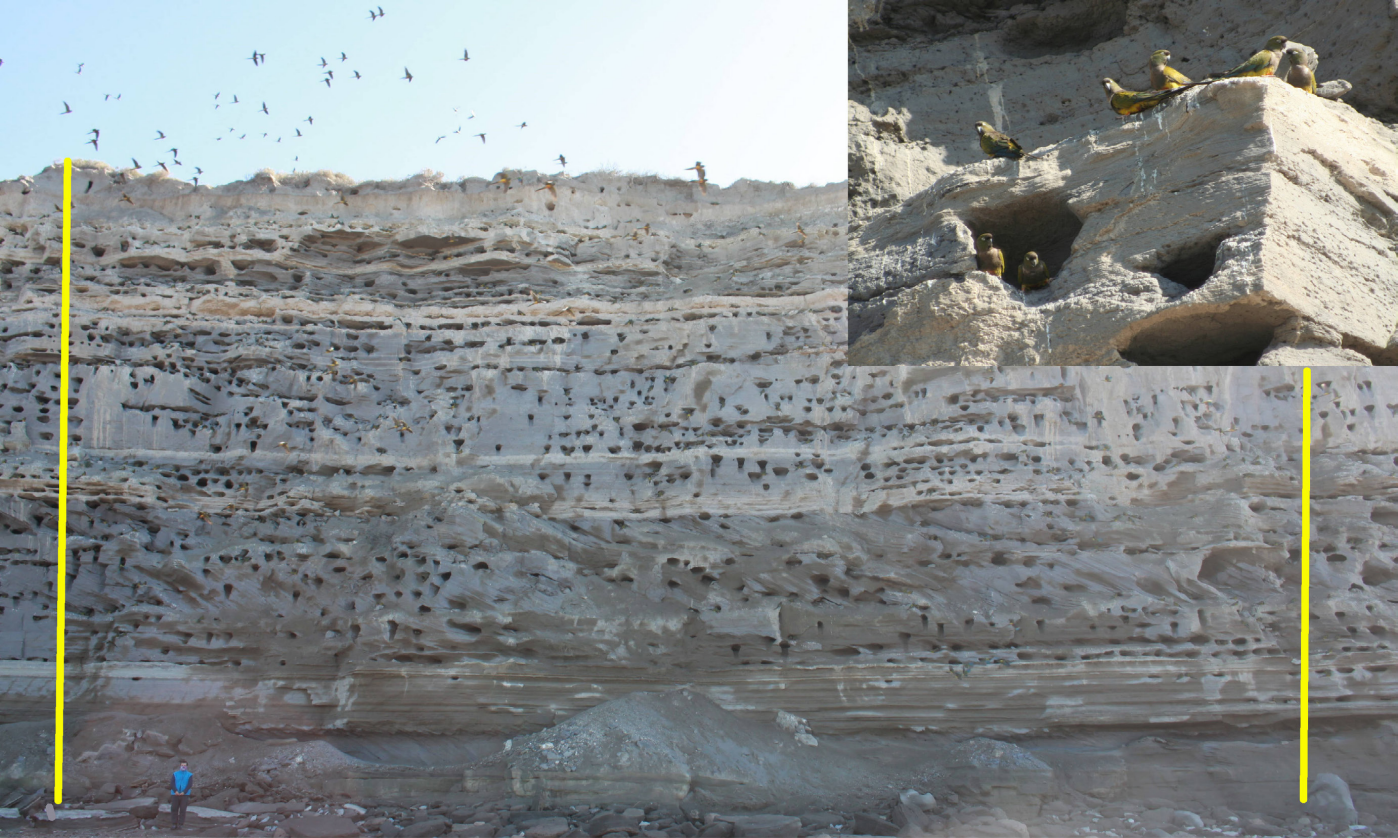
An example of a photograph taken to count nest entrances in a section of the cliff with the Burrowing Parrot *Cyanoliseus patagonus* colony at El Cóndor, province of Río Negro, Patagonia, Argentina. Numerous nest entrances can be seen in this image, as well as a flock of Burrowing Parrots at the top left. As a size reference, the person in the photograph is 170 cm tall. The yellow lines delimit, to the right and the left, the part of the image that was unique i.e., not duplicated in previous of following photograph in the array. *Inset*: A detail of the nests, with a pair of Burrowing Parrots standing at a nest entrance (photograph: Alejandro Balbiano).

The Burrowing Parrot (*Cyanoliseus patagonus*) is a Neotropical Psittaciformes (Aves) from the arid to semi‐arid shrub regions of Argentina and Chile (Masello et al., 2011, 2015). They are colonial birds that use soft stones or earth cliffs, where the birds excavate their nests. Burrowing Parrot breeding colonies, particularly in Argentina, greatly vary in size, with the great majority of them ranging from 5 to 420 nests (Masello et al., 2011, 2015). Following the cliff stratification, Burrowing Parrot breeding pairs dig their burrows in the softest layers, which vary from 0.6 m to more than 3.5 m deep (Masello et al., 2001, 2006). The nest entrances are approximately elliptical in shape, with the major axis horizontal (mean width 26.4 cm) and the minor axis vertical (mean height 12.9 cm; Masello et al., 2006; see also inset in Figure 1). Breeding pairs repeatedly use the burrow that they have dug and deepened, in previous seasons (Masello et al., 2006; Masello & Quillfeldt, 2002). Only one pair occupies each burrow (Masello & Quillfeldt, 2002).

The largest Burrowing Parrot colony is located on a cliff facing the Atlantic Ocean, adjacent to the village of El Cóndor, which is close to the estuary of the Río Negro, in north‐eastern Patagonia, Argentina (Masello et al., 2006). The 30 to 37 m high cliff is composed mainly of sandstone belonging to the Río Negro Formation (Angulo & Casamiquela, 1982; del Río et al., 2005). Though the westernmost part of the cliff contains a compact layer of clay at the bottom, which is not used by the parrots, and layers of soft sandstone on top (Masello et al., 2006). Previously, we estimated the number of Burrowing Parrot nests by means of manual counts from photographs performed by two trained persons (Masello et al., 2006). This task was very time‐consuming given the huge size of the Burrowing Parrot colony at El Cóndor. Nest entrances are mostly easily identified on photographs (Figure 1), but, sometimes, natural holes produced by erosion can be confused with parrot nests. Using that method, we found that the colony comprised 51,412 burrows and spread uninterruptedly over the first 9 km of the cliff (Masello et al., 2006). By climbing the cliff and directly inspecting the burrows dug by the Burrowing Parrots, we found that 3.8% of the burrows had two entrances leading to a single nest chamber, 10% of the nests were collapsed, and 73% of the nests were active in an intensively studied sector of the colony. Previous work (Masello et al., 2006) demonstrated that the number of nest entrances, in combination with the proportion of nests with two entrances, the proportion of collapse nests, and the proportion of active nests, provide a good estimation of population size. Using all this information, we estimated that the colony contained 37,527 active nests during the breeding season 2001–2002 (Masello et al., 2006). A later study showed that the colony grew further to the west, reaching a length of 12.5 km at the end of the 2000s (Llanos et al., 2011), a process that is continuing today. In December 2021, we measured a colony extent of 18.1 km. El Cóndor colony is of utmost importance for the conservation of Burrowing Parrots: 71% of the global population breeds at El Cóndor (Masello et al., 2011; Masello & Quillfeldt, 2012). Consequently, any threats affecting the El Cóndor colony could utterly impact the whole of the species. Furthermore, this colony is also deemed the largest known Psittaciformes colony in the world (Masello et al., 2006). Yet, it is seriously threatened by the strong clearance of the surrounding Monte vegetation and increasing urbanization in the vicinity of the cliff with the colony and on top of it (Masello et al., 2011; Masello & Balbiano, 2022; Masello & Quillfeldt, 2012).

In this study, we took advantage of convolutional neural networks to count the number of nest‐entrances from photographs taken across the El Cóndor Burrowing Parrot colony. We further aimed to detect changes in the number of nest‐entrances comparing the current count to our previous manual one and to better describe the distribution of nest entrances along the now longer colony. With all this, we further aimed to contribute information that may help to the conservation of this outstanding colony of Psittaciformes.

## 2 MATERIALS AND METHODS

### 2.1 Data collection

To count the total number of nest‐entrances, we took 222 photographs (3000 × 4000 pixels) across the entire colony during the breeding season 2019–2020 (Tables 1, 2, 3). To make the results of this study comparable to those from Masello et al. (2006) and to allow a detailed description of the distribution of burrows along the colony, we also recorded the GPS position of the start of each kilometre of the colony. As in Masello et al. (2006), we call ‘kilometre 1’ the easternmost kilometre of the El Cóndor colony, with the kilometre numeration progressing towards the westernmost kilometre of the colony (‘kilometre 18’).

**TABLE 1:** Parameters corresponding to the seven different trainings of the U‐Net architecture, errors of the seven models obtained, and nest‐entrance counts per kilometre of the Burrowing Parrot *Cyanoliseus patagonus* colony at El Cóndor, Río Negro, Argentina.

	Run							Mean ± SD
	1	2	3	4	5	6	7	
Epochs	80	100	100	100	90	90	100	–
Batch size	4	4	4	4	4	4	4	–
Training loss^a^	0.476	0.467	0.477	0.556	0.491	0.512	0.5	0.497 ± 0.30
Training *R* ^2^ ^a^	.97	.98	.98	.96	.98	.97	.97	.97 ± .01
Validation loss	2.520	2.813	3.184	2.255	2.649	2.365	2.6	2.627 ± 0.307
Validation *R* ^2^	.67	.64	.60	.53	.63	(−)	(−)	.61 ± .05
Mean absolute error^b^	3.4	4.2	1.7	2.9	2.1	2.7	1.9	2.7 ± 0.9
Maximum absolute error^b^	13.9	21.6	8.6	7.5	10.3	14.7	12.7	–

Nest‐entrance counts								Nr. photographs
Kilometre 1	6625.3	6722.8	4967.1	8809.1	6384.8	6218.6	5938.7	29
Kilometre 2	5704.8	4848.5	4721.5	6714.2	4364.4	5003.4	5066.5	10
Kilometre 3	8187.6	6907.3	7503.6	9590.0	7069.2	7548.4	7623.0	10
Kilometre 4	5237.4	4624.1	4917.8	6153.9	4950.5	4969.1	5015.9	6
Kilometre 5	6186.6	6004.0	5362.8	6594.0	5286.0	5989.0	5494.4	10
Kilometre 6	9896.7	9741.3	8099.0	10,671.9	8237.2	9816.1	8314.0	40
Kilometre 7	5974.5	5980.3	5287.2	6416.8	4797.8	6079.0	5264.3	18
Kilometre 8	4996.2	4731.4	4725.2	5451.1	4240.7	5018.8	4550.2	14
Kilometre 9	2064.6	2012.2	1864.0	2327.7	1627.3	2118.8	1787.4	10
Kilometre 10	2843.0	2885.1	2637.7	2902.0	2427.3	2841.3	2366.4	15
Kilometre 11	666.5	691.0	627.3	892.0	611.7	662.0	564.2	9
Kilometre 12	165.0	199.0	181.5	271.7	151.8	151.2	179.5	6
Kilometre 13	412.0	406.5	520.3	534.1	454.5	442.3	425.9	5
Kilometre 14	586.6	531.1	1137.1	1100.3	726.2	610.4	750.1	7
Kilometre 15	66.1	189.4	167.8	264.5	127.9	208.3	136.5	4
Kilometre 16	1285.3	1288.0	965.9	1408.7	1052.6	1133.0	961.0	16
Kilometre 17	1711.3	1442.0	1269.0	1663.7	1334.2	1408.3	1280.0	12
Kilometre 18	92.2	93.2	61.1	105.5	72.5	80.7	71.7	1^c^
Total colony	62,701.7	59,297.2	55,015.9	71,871.2	53,916.6	60,298.7	55,789.7	222
Mean total colony	59,842							

*Note*: (−) denotes a collapse during the entire training.

^a^ Losses and *R* ^2^ correspond to the last training epoch.

^b^ Over the testing patches.

^c^ During the breeding season 2019–2020, only the first 100 m of ‘kilometre 18’ (see Section 2) contained Burrowing Parrot nests, and, for that reason, we only took one photograph.

We took photographs from the beach that forms at the bottom of the cliff with the Burrowing Parrot colony during low tide. The beach can only be accessed from its easternmost part (kilometre 1) and using pedestrian and car accesses located at the start of kilometre 2 (called ‘Segunda Bajada del Faro’), in kilometre 15 (‘El Espigón’), and at the start of kilometre 17 (‘Playa Bonita’). This, combined with the fact that the ocean waters reach the bottom of the cliff during high tide, limited the accessibility of the colony during fieldwork, and imposed restrictions on when the fieldwork could be done. Likewise, the number of photographs per kilometre of the colony varied depending on the tides during fieldwork (Tables 1, 2, 3). During low tide, one of us (MF) was able to take photographs from further away from the cliff, which resulted in a larger portion of the cliff included per photograph (about 200 m). As high tide approached, the photographer was forced to take photographs closer to the cliff, thus capturing shorter portions of it (about 50 m). As during the high tide it was not possible to take photographs, and given the considerable size of the Burrowing Parrot colony at El Cóndor, we were forced to take the photographs over a period of 4 days. Consequently, the photographs show small differences in illumination. To reduce the differences in illumination, we chose cloudy days to avoid too strong shadows. According to our experience with manual counts (Masello et al., 2006), shadows cause difficulties in the counting of the nest entrances.

### 2.2 Pre‐processing and training

Consecutive photographs overlapped slightly with each other. In order to avoid counting nests twice, we marked each photograph with two vertical yellow lines delimiting, to the right and the left, the part of the image that was unique (Figure 1). In the following steps, only the unique part of each photograph was used. We added a line‐detector to the counting algorithms (see below) to allow them the identification of the unique parts of each photograph. However, in some photographs, we detected other lines close to the horizontal than the delimiting yellow lines, which were due to background noise in the images related to the cliff lithology. To solve this problem, we calculated the slope of the lines and identified the yellow delimiting lines as those that were close to the vertical. We eliminated the lines with a tangent less than 5, which correspond to angles less than 78.7°.

**FIGURE 2:**
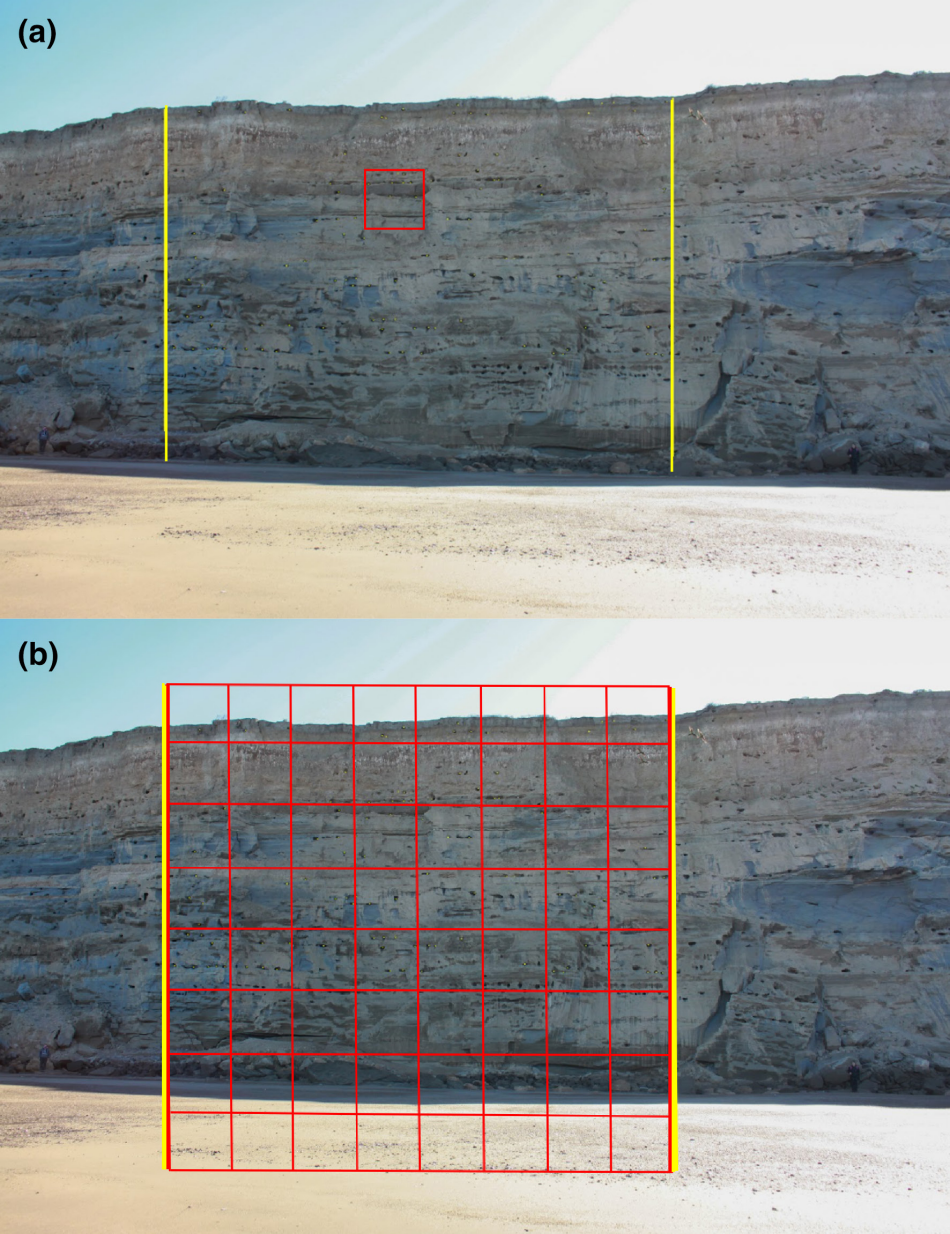
Example of one of the 222 photographs we took to cover the entire cliff with the Burrowing Parrot colony at El Cóndor, where we (a) selected a 256 × 256 pixels training patch, and (b) gridded the part of the image that is unique i.e. not duplicated in the previous or following photographs (between the yellow lines). Consecutive photographs in the series covering the entire colony overlap slightly to each other to ensure that all nest entrances were recorded.

**FIGURE 3:**
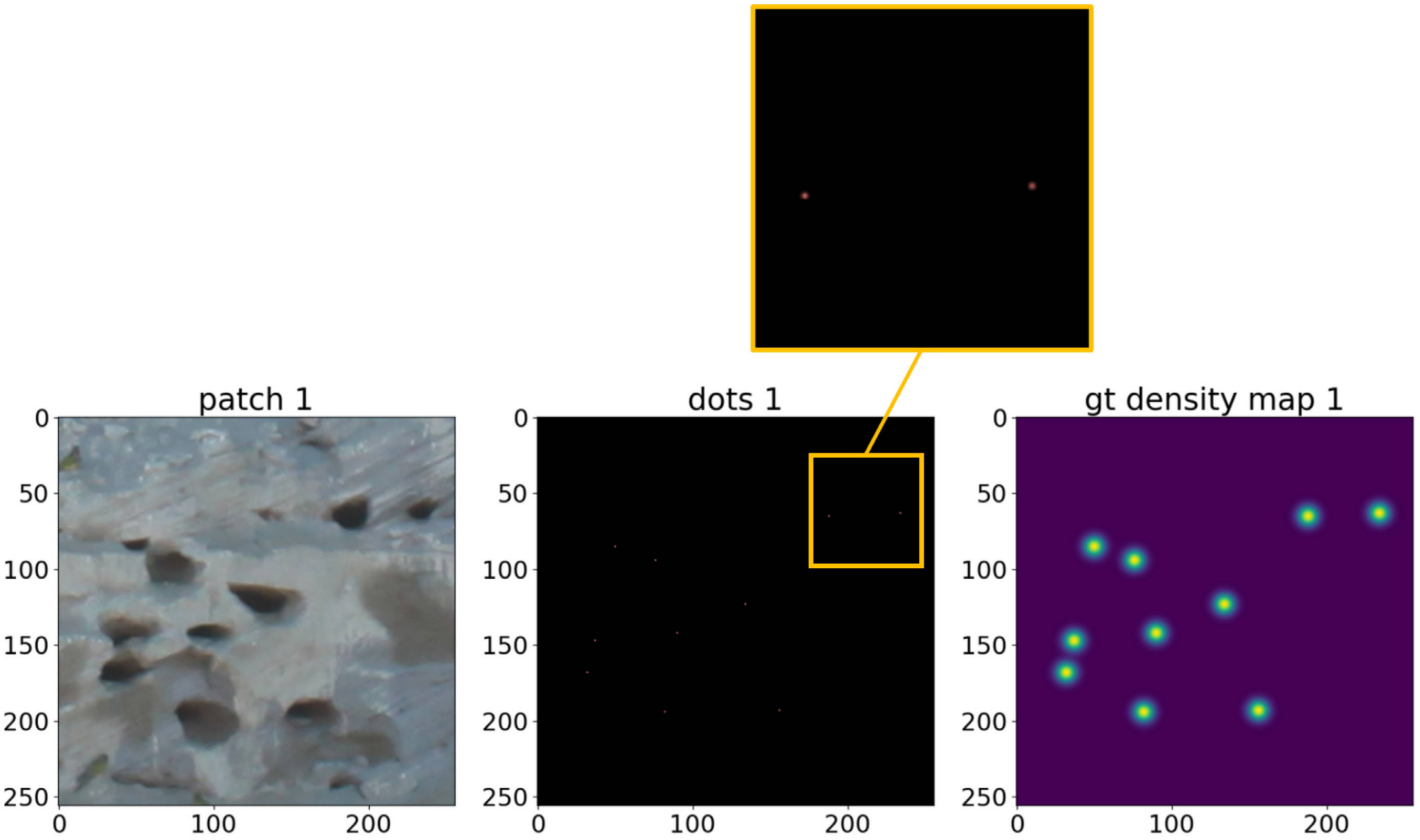
Illustration of the process followed to obtain ground truth density maps. In a patch of 256 × 256 pixels that we randomly cut from the original photographs (patch 1; see Figure 2), each nest is annotated with a single pixel (dots 1; the inset shows two dots in detail). In a following step, a Gaussian blur on the dots image produces the density map (gt density map 1).

Our analyses are based on the methodology developed by Lempitsky and Zisserman (2010) for visual object counts. This supervised learning framework, suitable for any domain‐specific visual features, is trained to accurately predict object density maps from input images (Lempitsky & Zisserman, 2010). Once the inferred density maps are obtained, the object count is achieved through a subsequent mathematical integration process that simply sums the pixel values of the density maps. The process of learning to infer such density involves minimizing a regularized risk quadratic cost function (Fiaschi et al., 2012; Lempitsky & Zisserman, 2010; Xie et al., 2018). This method requires a set of training images with annotation, but instead of using bounding‐box or pixel‐accurate annotation, it takes advantage of dotted supervision (Lempitsky & Zisserman, 2010). In this case, the object instances present in the training images are annotated using a single dot on each of them. According to Lempitsky and Zisserman (2010), dotted annotation is the natural way to count objects for humans, is less labour‐intensive, and offers additional spatial information than traditional bounding‐box or pixel‐accurate annotations. Such training images, augmented with dotted annotations, allow the framework developed by Lempitsky and Zisserman (2010) to learn to count the number of object instances in previously unseen images. To produce the ground‐truthed density maps necessary to train the CNN for pattern recognition, we selected 156 patches (also called ‘regions’ in Lempitsky & Zisserman, 2010) of 256 × 256 pixels (e.g. Figure 2a). We randomly selected those training patches from the original photographs (e.g. Figure 2a), using an image manipulation program (GIMP 2.8.10, Spencer Kimball, Peter Mattis and the GIMP Development Team), and saved them as raster‐graphics files (portable network graphics, png). The training patches included parts of the cliff with nests that we photographed under slightly different light conditions or distances to the cliff to account for variability in the quality of the original photographs. The patches taken for training purposes were much fewer and, to improve generalization, did not coincide in position with subsequent patches generated along the grid, needed to infer density maps prior to the full colony counting process. The amount of training patches we produced represents a compromise between the time invested to annotate them and the quality of the results obtained during CNN testing runs. As the next step, one of us, with extensive experience in counting Burrowing Parrot nest‐entrances from photographs (JFM; Masello et al., 2006, 2011), fully annotated those training patches by manually marking the approximate centre of each nest‐entrance with a 1 × 1 pixel dot, also using GIMP 2.8.10 (Figure 3, dots 1). Subsequently, we created a new png image, composed of a uniform background and the annotation single dots, which spatially locate the nest‐entrances (Figure 3, dots 1), following the methodology by Lempitsky and Zisserman (2010). In this way, we were able to use the spatial information contained in the images, with the count of the annotation dots representing the actual count of the object instances (nest‐entrances in our particular case) in the photographs. We used red annotation dots and a black background (Figure 3), yet other colours could have been used for the same purpose (i.e. to produce single dots and uniform backgrounds). We additionally performed a geometric data augmentation by applying horizontal flipping, since the approximately elliptical shape of the nest‐entrances did not allow for performing vertical flipping. Horizontal flipping has been shown to enhance the size and quality of training datasets (Shorten & Khoshgoftaar, 2019). This way, we were able to double the total number of training patches from 156 to 312. Finally, we blurred the points using a Gaussian convolution (also, called Gaussian blur or smoothing; Chandel & Gupta, 2013; Gedraite & Hadad, 2011) to create ground‐truthed density maps (Figure 3, gt density map 1). When the expert biologist annotates the approximate centre of the nest entrances, it uses one pixel of value 1 per nest entrance. When applying a Gaussian convolution in the vicinity of an isolated pixel with a value of 1, the value spreads out to its neighbouring pixels, creating a blurred effect. The pixel itself retains the highest value, while the values of neighbouring pixels decrease with distance, forming a smooth, bell‐shaped curve centred on the original pixel. This results in a gradient of values that smoothly transitions from the central pixel outward. All the pixels in this area now have values lower than 1. The pixels furthest from the centre of this bell have very low values, much less than 1. Following Waithe (2017), we scaled the density maps from 0.0–1.0 to 0.0–255.0, otherwise, the network struggles to learn the very small target values produced by the Gaussian convolution. We subsequently downscaled those values to valid values of density, 0.0–1.0 (Waithe, 2017). To improve the generalization performance of the model, we split the data into training, validation, and test sets. This serves the purpose of training the model on the training set, tuning the hyperparameters using the validation set, and assessing the performance of the final model on the test set. The goal of the training process is to achieve a level of generalization that allows the model to effectively handle new and unseen data in the validation and test sets. For good generalization, it is important to ensure that the training, validation, and test sets have similar statistical distributions (Borovicka et al., 2012). Although there are no definitive splitting suggestions and each case must be examined individually, it is typical to use an 80/10/10 distribution, randomly selecting 80% training, 10% validation, and 10% testing. However, a larger proportion of data allocated for training facilitates the model to learn more complex relationships, and a smaller test set suffices if it reflects the statistical distribution of the training set. To approximate this similarity between sets in the statistical distribution, we performed seven different trainings for each of the three architectures used (U‐Net, ResUnet, and DeepLabv3; Chen et al., 2018; Ronneberger et al., 2015; Zhang et al., 2018), randomly shuffling the 312 patches and splitting them into 256 patches for training, 40 patches for validation, and 16 testing patches each time. Maintaining the same order of original image patches and corresponding ground‐truthed patches during shuffling and splitting is mandatory. The results from each of the seven runs were then averaged to provide a final estimate of model performance.

### 2.3 Algorithms used and nest counts

We used three different CNN architectures, U‐Net, ResUnet, and DeepLabv3 (Chen et al., 2018; Ronneberger et al., 2015; Zhang et al., 2018), to predict density maps, which we then mathematically integrated to count the total number of nest‐entrances in the 222 photographs we took across the entire cliff with the Burrowing Parrot colony at El Cóndor (Tables 1, 2, 3).

The U‐Net architecture was originally developed to segmentate biomedical images (Ronneberger et al., 2015). We adapted this CNN to regress the required density maps (Waithe, 2017; Zanellato, 2021). The object count was derived by integrating the intensities of the pixels over the density maps. The U‐Net architecture is symmetric and includes an encoder, a bottleneck, and a decoder with skip connections that copy maps from the encoder to the decoder, enhancing network performance by preserving information. To build the network, we followed Paul (2018), using an input size of 256 × 256 pixels, three RGB channels, rectified linear unit activation (Relu; Lin & Shen, 2018), the same padding, and a dropout of 15% in the last two blocks of the encoder, totalling 31,032,837 parameters. For the compilation, we employed the Adam optimizer (Kingma & Ba, 2015) with an initial learning rate 1e^−4^, a loss function rmse (root mean square error; Hodson, 2022), and *R* ^2^ as the training metric. rmse quantifies the prediction errors, weighting larger errors more heavily (Hodson, 2022). Backpropagation computes the gradients of the loss function to update the parameters in the direction that minimizes the loss (LeCun et al., 1998). The Adam optimizer adapts the learning rates for efficient training. We trained the network for 100 epochs with a batch size of four using the GPUs available on the Google Colaboratory platform (http://colab.research.google.com/ accessed 11 April 2024), typically Tesla T4. Some runs were executed with the pro‐option, V100 or A100 Nvidia GPU.

The ResUnet combines residual learning with U‐Net for image segmentation and density map regression. Adapted from Zhang et al. (2018), it employs residual units in a U‐Net‐like architecture. We used the nikhilroxtomar repository on GitHub (Tomar, 2019) for the implementation.

DeepLabv3 segments objects at multiple scales by adjusting the filter's field‐of‐view and controlling feature resolution with varying convolution rates (Chen et al., 2018). Using the nikhilroxtomar repository (Tomar, 2021), we adapted this network, initially developed for human image segmentation, to regress density maps. The encoder uses ResNet50 with “Imagenet” weights available in the Tensorflow Keras library (https://keras.io/api/applications/; Krizhevsky et al., 2017; Zeiler & Fergus, 2014) and implements Atrous Spatial Pyramid Pooling (ASPP) with atrous convolutions at different rates to encode objects at multiple scales (Chen et al., 2017). The activation maps were up sampled to match the input image size.

The implemented architectures typically use sigmoid activation at the network output (Tomar, 2019), with a binary mask with pixel value 1 and a background with pixel value 0, which constrains the sigmoid function output values to 0.0–1.0. After following Waithe (2017) recommendation, increasing the density values in the ground‐truthed patches from 0.0–1.0 to 0.0–255.0, we needed an adequate activation function. We selected the Relu activation function, which is not constrained in the positive range of the ordinate axis (Lin & Shen, 2018). Subsequently, we descaled the predicted density maps to the 0.0–1.0 range before counting.

**FIGURE 4:**
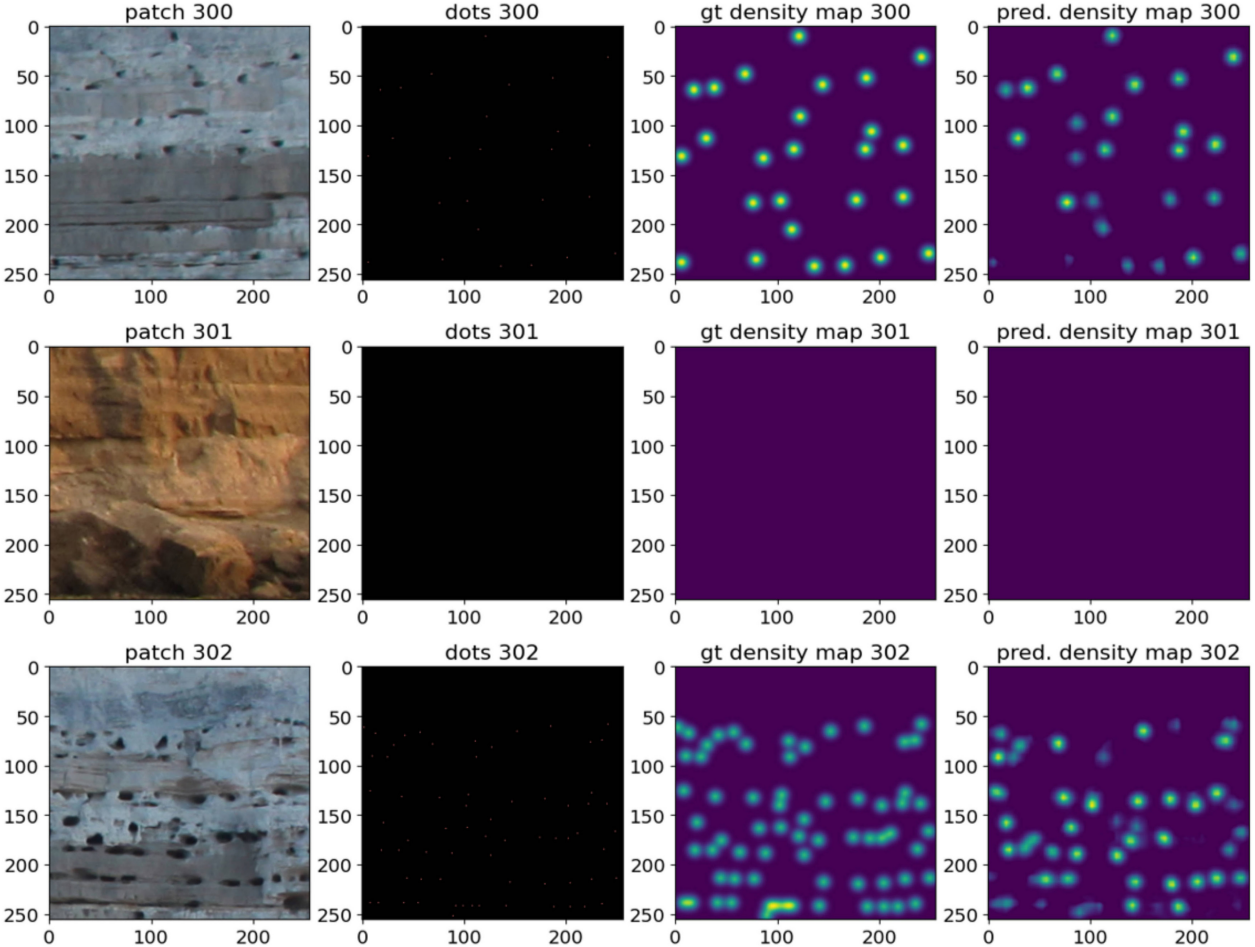
Three different examples of the testing set, where we compare the original training patch, the dots patch, the ground truth density map, and the predicted density map. Particularly, patch 301 is one of those added to make the algorithm learn not to count where there are no nests.

After the training phase, we tested the models over the randomly selected 16 testing patches, plotted the predicted density maps, and counted the objects (Figure 4). For each of the testing patches, we calculated the count error, that is, the difference between the actual nest count and the predicted nest count. The actual nest‐entrance count is simply the sum of all individual pixels with value one, which represents the centre of each nest‐entrance in the dotted training patches. The predicted nest‐entrance count is the sum of the pixel values of the entire predicted density map, after the aforementioned downscale of pixel intensities to the range 0.0–1.0. In some cases, the count error is positive, in others it is negative. Next, we computed the mean absolute error (MAE) of the 16 test patches.

Training models on different data shuffles can provide more robust estimates of model performance and help better understand the variability of the results (Haochen & Sra, 2019). Thus, we decided to train seven different models for each of the three architectures, balancing the available time and resources with the quality of results. To perform seven different trainings for each of the CNN architectures used, we changed the seed for randomly selecting the train, validation and test patches over the total available 312 patches. During the training, we tracked the train and validation losses, and the train and validation *R* ^2^. For the U‐Net architecture, after the total number of epochs, we reached train *R* ^2^ above .96 for all cases, and a minimum‐median‐maximum range of validation *R* ^2^ of .53–.63–.67. For the ResUnet architecture, the reached train *R* ^2^ was above .97 for all cases, and the corresponding range of validation *R* ^2^ was .43–.49–.58. Finally, for the DeepLabv3 architecture, after the total number of epochs, the train *R* ^2^ was above .97 for all cases, and the same range of validation *R* ^2^ was .53–.63–.69.

**FIGURE 5:**
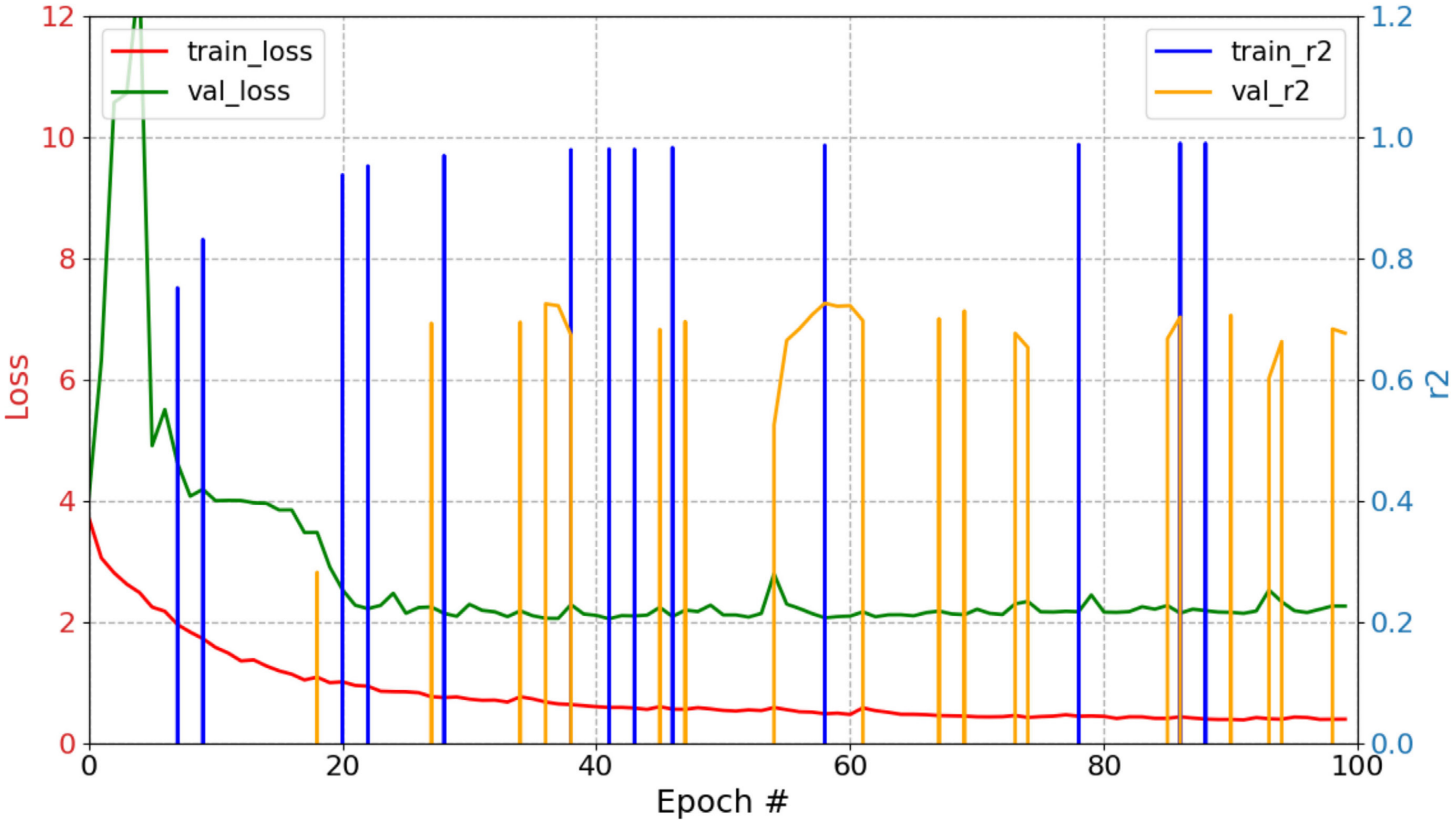
Example of training curves where we can see training and validation losses and *R* ^2^. Most of the time *R* ^2^ collapses to negative numbers, however loss trends always decrease.

Finally, we gridded the 222 original photographs, covering the entire Burrowing Parrot colony, into 256 × 256‐pixel patches, as shown in Figure 2b, passed them through the model, and ran the nest‐entrance counts. But, in the first stage of our work, when we started training the models only with patches that contained nest entrances, when plotting predicted density maps and counting objects on them, we observed a noticeable overcounting of nest entrances. The algorithm was detecting false positives in several patches corresponding to sectors of the cliff with no nest‐entrances and patches that included the sky above the cliff or the beach that forms below the cliff during low tide. To correct this issue, we added to the training set patches belonging to those areas (40% of the patches), where the ground‐truthed map was an empty map (e.g. patch 301 in Figure 4). This action affected the *R* ^2^ curves of the training, which sometimes collapsed to very large negative values (Figure 5). According to the *R* ^2^ equation, this is a certain possibility if the predictions are very inaccurate, that is, the actual values are very different from the predicted values. The sum of the squared difference in the numerator of the second term is preceded by a negative sign:

$$R^{2}=1-\frac{\sum_{i=1}^{n}{\left(\mathrm{yi}-\hat{y}i\right)}^{2}}{\sum_{i=1}^{n}{\left(\mathrm{yi}-\bar{y}i\right)}^{2}}$$

where $y_{i}$ denotes the actual values, ${\hat{y}}_{i}$ the predicted values, and ${\bar{y}}_{i}$ the arithmetic means of the class. *R* ^2^ = 1 describes a perfect prediction, while *R* ^2^ = 0 implies that the model delivers the arithmetic mean as prediction. This is why a validation *R* ^2^ around .5, like the one reported for ResUnet, is actually an adequate one. *R* ^2^ is used to assess how well the independent variables in a regression model explain the variability observed in the dependent variable. It is a useful metric for evaluating the goodness of fit of the model to the data (Draper & Smith, 1998). What is really important are the values and trend of the loss curves since they are what change the weights of the network. If the validation loss curve shows an increasing trend from a certain point, we are facing an overfitting problem. In our case, however, the losses were always minimized, and *R* ^2^, when positive, increased, the training proceeded, and the overcounting was solved. We never observed conspicuous overfitting in our training.

We checked for deviations from normality with Kolmogorov‐Smirnov tests. In cases where data were not normally distributed, we provide medians and ranges. We split the nest counts per kilometre of the colony to allow comparisons with our previous study and to detect possible changes in the distribution of nests along the Burrowing Parrot colony (Masello et al., 2006).

## 3 RESULTS

On the 222 photographs across El Cóndor Burrowing Parrot colony, the U‐Net architecture counted a mean of 59,842 (range, 53,916.6–71,871.2) nest‐entrances (Table 1), while DeepLabv3 counted 46,193 (35,943.0–53,605.9; Table 2), and ResUnet 52,863 (43,210.4–71,158.2; Table 3). We found that the U‐Net architecture had the lowest mean error (2.7 nest‐entrances, range, 1.7–4.2) and maximum absolute error (21.6 objects in one patch) over the testing patches (Table 1). For DeepLabv3, the mean of the error was 3.6 nest‐entrances (range 2.4–4.9), with a maximum absolute error of 29.7 objects in one patch (Table 2), while for ResUnet, the mean error was 4.1 nest‐entrances (range, 1.8–7.9), with a maximum absolute error of 30 objects in one patch (Table 3).

**TABLE 2:** Parameters corresponding to the seven different trainings of the DeepLabv3 architecture, errors of the seven models obtained, and nest‐entrance counts per kilometre of the Burrowing Parrot *Cyanoliseus patagonus* colony at El Cóndor, Río Negro, Argentina.

	Run							Mean ± SD^a^
	1	2	3	4	5	6	7	
Epochs	100	100	100	100	100	100	100	–
Batch size	4	4	4	4	4	4	4	–
Training loss^b^	0.387	0.367	0.366	0.388	0.378	0.350	0.403	0.377 ± 0.018
Training *R* ^2^ ^b^	.99	.99	.99	.99	(−)	.99	.99	.99
Validation loss	2.227	2.236	2.252	2.362	2.485	2.399	2.483	2.349 ± 0.113
Validation *R* ^2^	(−)	(−)	.686	.682	.574	(−)	.530	.618 ± 0.078
Mean absolute error^c^	2.7	2.4	4.6	3.3	4.9	3.3	4	3.6 ± 0.94
Maximum absolute error^c^	22.3	22.8	29.2	15.9	19	12.6	29.7	–

Nest‐entrance counts								Nr. photographs
Kilometre 1	6509	9334.2	4924.2	6677.2	7477	5344.3	7739.8	29
Kilometre 2	3551.1	4367.7	2726.6	4922.6	4056.4	3768.8	3564.9	10
Kilometre 3	5027.9	5785.5	3933.1	7136.6	5079.4	5666.7	4457.2	10
Kilometre 4	3077.6	3785.6	2567.4	5023.4	3023.6	3540.9	2968.3	6
Kilometre 5	4331.6	4958.7	3777.9	5232.2	4778.3	4892.9	4491.1	10
Kilometre 6	6309.2	7929.4	6165.6	6789.7	7860.4	6846.1	6663.8	40
Kilometre 7	4549	5375.8	4171.3	4398.8	5250.4	4858.1	4373.3	18
Kilometre 8	3243.6	3924.5	3043.4	3488.7	4101.9	3714.6	3133.6	14
Kilometre 9	1532.9	1790.6	1431.6	1455	2025.5	1720.5	1420.3	10
Kilometre 10	1595.3	2280.5	1503.1	1488.5	2207.6	1747.4	1718.5	15
Kilometre 11	353.9	675.9	278.1	2020.1	1197.2	487.3	374.2	9
Kilometre 12	142.2	309.7	108.3	1053.2	824.5	140.8	179.5	6
Kilometre 13	219.6	458.1	157.3	695.7	525.7	312.7	207.7	5
Kilometre 14	299.9	617.9	152.8	521.6	624.8	417.7	261.6	7
Kilometre 15	165.2	315.1	81.6	210.5	366.2	188.2	98.8	4
Kilometre 16	528.3	849.8	451.1	1219.5	830.3	638.1	577.8	16
Kilometre 17	530.7	769.3	429.3	646.6	718	608.8	422.4	12
Kilometre 18	55.1	77.6	40.3	142.1	69.3	52.8	40.4	1^d^
Total colony	42,022.1	53,605.9	35,943	53,122	51,016.5	44,946.7	42,693.2	222
Mean total colony	46,193							

*Note*: (−) denotes a collapse during the entire training.

^a^ If not normally distributed, only the median is provided.

^b^ Losses and *R* ^2^ correspond to the last training epoch.

^c^ Over the testing patches.

^d^ During the breeding season 2019–2020, only the first 100 m of ‘kilometre 18’ (see Section 2) contained Burrowing Parrot nests, and, for that reason, we only took one photograph.

**TABLE 3:** Parameters corresponding to the seven different trainings of the ResUnet architecture, errors of the seven models obtained, and nest‐entrance counts per kilometre of the Burrowing Parrot *Cyanoliseus patagonus* colony at El Cóndor, Río Negro, Argentina.

	Run							Mean ± SD^a^
	1	2	3	4	5	6	7	
Epochs	80	80	80	80	80	80	80	–
Batch size	4	4	4	4	4	4	4	–
Training loss^b^	0.451	0.513	0.789	0.407	0.389	0.437	0.448	0.448
Training *R* ^2^ ^b^	.98	.97	.97	.98	.98	.99	.98	.99
Validation loss	3.520	2.934	2.686	2.800	3.830	2.656	2.888	2.888
Validation *R* ^2^	(−)	.48	.49	.58	.50	.43	(−)	.494 ± .064
Mean absolute error^c^	3.4	4.6	3.8	1.8	4.6	2.5	7.9	4.1 ± 0.3
Maximum absolute error^c^	18.5	14.2	10.2	9.3	17.1	7.5	30	–

Nest‐entrance counts								Nr. photographs
Kilometre 1	4153.8	5141	9267.9	6456.7	5527.5	8069	5313.1	29
Kilometre 2	3805.2	3980.4	6079.7	4740.6	3900	5757.9	4121	10
Kilometre 3	5340.9	5361.9	8113.8	6143.3	5085.8	7672.7	4974.6	10
Kilometre 4	3593	3447.3	5266.3	4112	3615.2	4986.4	3374.1	6
Kilometre 5	4276.3	4144	6204.5	5051.6	4710.8	5847.1	4315.5	10
Kilometre 6	6975.2	7691.5	10,939.8	8222.7	7080.4	10,513	7017.8	40
Kilometre 7	4062.6	4728	6155.5	5089.8	3813.7	6148.2	3933.1	18
Kilometre 8	3757.3	4203.7	5602.5	4403.6	3370.8	5535.9	3503.5	14
Kilometre 9	1498.8	1793.7	2360	1878.6	1295.9	2269.5	1472.7	10
Kilometre 10	2124	2790.5	4011	2923.8	2017.2	3757.7	2113.4	15
Kilometre 11	544.6	687.4	1142.1	663.8	582.3	1044.6	547	9
Kilometre 12	125.1	163.5	370.4	172.2	140.6	283.8	139.8	6
Kilometre 13	369.1	448.4	745.4	456.1	361.5	662.5	369.3	5
Kilometre 14	387.1	473.8	813.7	427.7	308.5	710	362.2	7
Kilometre 15	127.1	92.8	209.2	91.7	86.9	197.9	77.5	4
Kilometre 16	894.3	958.7	1718.9	1071.3	1074.7	1670.7	921.6	16
Kilometre 17	1100.1	1124.7	1991.3	1265.2	1251.7	1874.5	1008.5	12
Kilometre 18	75.9	70.9	166.2	91.3	93.5	144.5	82.9	1^d^
Total colony	43,210.4	47,302.2	71,158.2	53,262	44,317	67,145.9	43,647.6	222
Mean total colony	52,863							

*Note*: (−) denotes a collapse during the entire training.

^a^ If not normally distributed, only the median is provided.

^b^ Losses and *R* ^2^ correspond to the last training epoch.

^c^ Over the testing patches.

^d^ During the breeding season 2019–2020, only the first 100 m of ‘kilometre 18’ (see Section 2) contained Burrowing Parrot nests, and, for that reason, we only took one photograph.

**FIGURE 6:**
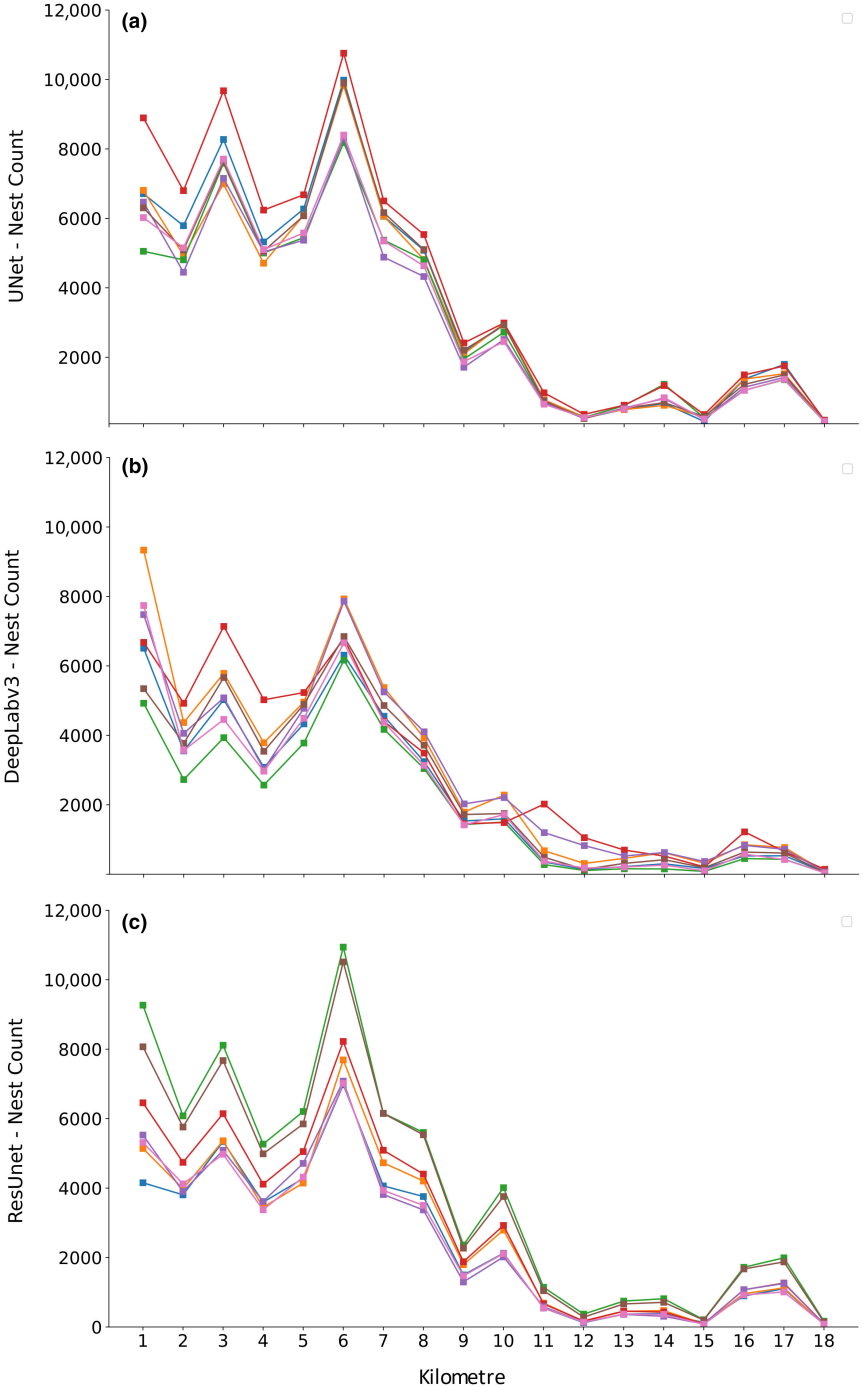
Burrowing Parrot *Cyanoliseus patagonus* nest counts per kilometre of the colony at El Cóndor, province of Río Negro, Patagonia, Argentina, (km 1 corresponds to the easternmost kilometre) resulting from the different models, run with the (a) U‐Net, (b) DeepLabv3, and (c) ResUnet architectures. For each architecture, the different models are colour‐coded.

We present the distribution of nest‐entrances across the Burrowing Parrot colony at El Cóndor in Figure 6. All models for all three CNN architectures used revealed the same pattern: higher number of nest‐entrances in kilometres 1 to 7, the highest mean number of nests‐entrances in kilometre 6, a relatively slow decline from kilometres 8 to 11, and low number of nest entrances from kilometres 12 to 18 (Figure 6a–c).

The U‐Net architecture showed the lowest variability among the models (Figure 6a). However, all three architectures exhibited higher variability in the first (easternmost) kilometres of the El Cóndor Burrowing Parrot colony, mainly up to kilometre 6. This feature was more evident in the DeepLabv3 and ResUnet architectures (Figure 6b,c), which had high variability in kilometres 1 to 4.

## 4 DISCUSSION

Using three different CNN architectures, we were able to count the total number of nest‐entrances in the 222 photographs taken at El Cóndor, covering the entire Burrowing Parrot colony. The U‐Net architecture, which showed the best performance, counted a mean of 59,842 nest‐entrances in line with previously published manual counts (Masello et al., 2006). They are also in line with more recent manual counts (62,913 nest‐entrances; JFM unpubl. data) carried out also on the same pictures from the breeding season 2019–2020. However, the U‐Net shows 8430 more nest‐entrances than in the counts previously published and 3071 less than in the most recent manual count. These differences may have multiple origins. First, the three CNN architectures showed higher variance in the first kilometres, mainly up to kilometre 6 (Figure 6). This behaviour was more evident for the DeepLabv3 architecture, which exhibited in general low variance, but in kilometres 1 to 4 showed high variance (Figure 6b). These errors most likely originate in the quality of the photographs, which we were forced to take at varying distances to the cliff, from different angles, and with changing light conditions. Yet, the low mean error of the U‐Net architecture opens the possibility of using this methodology to count nest‐entrances from photographs taken at other Burrowing Parrot colonies, or even from other birds with similar nesting habits (e.g. Petrescu, 1998; Wermundsen, 1998). Burrowing Parrots presently confront a precarious conservation situation, as shown by a strong reduction in colony sizes in parts of its distributional range (Masello et al., 2015). During a survey of Burrowing Parrot colonies, we were able to locate the most active colonies of the species (Masello et al., 2011). The methodology here presented could be used to monitor those colonies and to produce regularly updated population size information to inform conservation programmes.

The difference among the results of current counts and previously published ones could also be related to the change in the extent of the colony over the period 2001–2021. In 2001, the Burrowing Parrot nests were distributed over 9 km of the cliffs next to the village of El Cóndor (Masello et al., 2006), while in 2021, they were distributed over twice as much that extent: 18 km. Thus, current CNN counts may truly imply an increase in the number of nest‐entrances and consequently population size, not being the result of methodological errors. However, it is very important to mention that the number of nest‐entrances alone does not directly represent the Burrowing Parrot population size at El Cóndor. As we previously showed (Masello et al., 2006), the number of Burrowing Parrot active nests in a colony, which better reflects the population size, also depends on other parameters. Due to the soft substrates on which Burrowing Parrots dig their burrows (Masello et al., 2006; Ramirez‐Herranz et al., 2017), there is always a proportion of naturally collapsed nests in a colony. Collapsed nests can be distinguished with certainty only by direct inspection (JFM pers. observation). In El Cóndor, this proportion slightly increased from year to year, starting with 2% at the beginning of our records in 1998 (JFM unpubl. data). During the 2001–2002 breeding season, that proportion was of 10% (Masello et al., 2006). Therefore, the apparent increase in the number of nest‐entrances suggested from current U‐Net counts might be due to an increase in the number of collapsed nests that has forced the Burrowing Parrots to dig more burrows and not necessarily to an increase in the number of active nests. To test this alternative, or even complementary, explanation, we would need to know the proportion of active nests, an information that cannot be gained from the photographs used in this study. Future studies should also incorporate nest activity, which can be determined by direct inspection (Masello & Quillfeldt, 2002) or using video recording (Masello et al., 2001, 2006), allowing a good estimation of population size (Masello et al., 2006).

The CNN architectures also revealed changes in the distribution pattern of nest‐entrances in the Burrowing Parrot colony at El Cóndor (Figure 6). Yorio and Harris (1997) mentioned that, in 1990, the densest part of the colony was at kilometre 1 (according to our definition), a pattern that we also observed during the breeding season 1999–2000 (Masello & Quillfeldt, 2002). In the 2001–2002 breeding season, 4 km at the eastern end of the colony were the most densely populated, with much less nest‐entrances in the 5 km at the western end, and the highest number of nest‐entrances placed in the kilometre 2 (Figure 2 in Masello et al., 2006). In 2019–2020, all models for all three CNN architectures show that the highest number of Burrowing Parrot nests‐entrances is currently located in kilometre 6, and that the most densely populated part of the colony now extends until kilometre 11, reaching a total extent of 18 km (Figure 6). All these results show a displacement of nest‐entrance numbers from the eastern end towards the western end, which has been progressing from the year 2000 onwards. This displacement can be attributed to increasing levels of human disturbance in the eastern end of the colony. As first mentioned in Masello et al. (2006), the village of El Cóndor, which in 1998 extended up to ca. 400 m eastwards of the Burrowing Parrot colony, expanded towards the colony, with its last buildings situated at less than 30 m from the easternmost end of the colony since the year 2000. During the breeding season 2000–2001, a car park was built on top of the western end of kilometre 1. This parking place triggered a strong erosion process that affected a sector of the cliff located below and the Burrowing Parrot nests it contained. This may also be the reason why the proportion of collapsed nests increased from 2% in 1998 to 10% in December 2001 (Masello et al., 2006). At the same time, the car access that is located between kilometres 1 and 2 was enlarged, and commercial extraction of sand was carried on the beach below, in the vicinity of that car access (Masello et al., 2006). Intense poaching activity affected kilometre 1 of the colony during 2002–2003. Moreover, since 2001, intense paragliding has been carried out particularly during the holiday season (December–February), which in part overlaps with the Burrowing Parrot breeding season (October–December; Masello & Quillfeldt, 2002; Masello et al., 2006). Furthermore, in 2015–2016, a large Malvinas memorial, including several heavy structures, was built on top of the cliff in the centre of kilometre 1. The Malvinas memorial was expanded in 2017–2018. Lastly, in several periods since 1998, and most recently during 2018–2022, a pit located a few hundred metres away from the top of the cliff and along kilometre 1, was used as a garbage dump (now closed and relocated).

## 5 CONCLUSIONS AND PERSPECTIVES

Our results demonstrate that CNN, particularly the U‐Net architecture, provides a reliable tool for the ongoing long‐term monitoring of the number of nest‐entrances, from photographs, at the Burrowing Parrot colony at El Condor. It could also be used for counting nests at other Burrowing Parrot colonies, and nests of other colonial birds breeding on cliffs or on the ground, but this would need to be tested in future studies, using specific training data for specific locations.

In fact, the results here presented reinforce the need for continuing and improving, with the use of CNN, the monitoring at El Cóndor. The strong changes here observed in the distribution of nest‐entrances along the cliff at El Cóndor are in line with a long record of human‐induced disturbances. Given that the total number of nest‐entrances counted in 2001–2002 and in 2019–2020 are similar, they are unlikely related to population size changes. Those changes might actually suggest that human‐induced disturbance to the colony during the last 20 years has forced part of this parrot population to dig their burrows in previously unused parts of the cliff. On the one hand, this shows a degree of adaptation on the part of the Burrowing Parrot. On the other hand, as we mentioned previously (Masello et al., 2006), a compact clay layer in the westernmost part of the colony (Angulo & Casamiquela, 1982) is not suitable for the parrots to dig their burrows. In the future, this feature of the cliff may impose a limit to the capacity the parrots may display to a further increase in human‐induced disturbance. El Cóndor holds most of the breeding population of the entire species (71%; Masello & Quillfeldt, 2012, Masello et al., 2011), being also deemed the largest known Psittaciformes colony in the world (Masello et al., 2006). Thus, the threats currently affecting this colony, or future ones, could have serious consequences for the whole species, and thus, they should be promptly tackled with adequate conservation measures.

## AUTHOR CONTRIBUTIONS

**Gabriel L. Zanellato:** Conceptualization (equal); data curation (equal); formal analysis (equal); methodology (equal); resources (equal); software (equal); validation (equal); visualization (equal); writing – original draft (equal); writing – review and editing (equal). **Gabriel A. Pagnossin:** Conceptualization (equal); investigation (equal); writing – review and editing (equal). **Mauricio Failla:** Methodology (equal); resources (equal); writing – review and editing (equal). **Juan F. Masello:** Conceptualization (equal); data curation (equal); formal analysis (equal); investigation (equal); methodology (equal); project administration (equal); resources (equal); software (equal); supervision (equal); validation (equal); visualization (equal); writing – original draft (equal); writing – review and editing (equal).

## CONFLICT OF INTEREST STATEMENT

The authors declare that they have no known competing financial interests or personal relationships that could have appeared to influence the work reported in this paper.

## Data Availability

All codes used in this study are freely available at https://github.com/gzanellato‐ia/burrowing_parrot.
